# Systemic capillary leak syndrome associated with a rare abdominal and four-limb compartment syndrome: a case report

**DOI:** 10.1186/1752-1947-8-196

**Published:** 2014-06-16

**Authors:** Hayat Lamou, Jan-Peter Grassmann, Marcel Betsch, Michael Wild, Mohssen Hakimi, Joachim Windolf, Pascal Jungbluth

**Affiliations:** 1 Department of Trauma and Handsurgery, Heinrich Heine University Hospital Düsseldorf, Moorenstr. 5, 40225 Düsseldorf, Germany

**Keywords:** Systemic capillary leak syndrome, Compartment syndrome, Fasciotomy, Interdisciplinary treatment

## Abstract

**Introduction:**

Systemic capillary leak syndrome is a rare and life threatening disease characterized by periodic episodes of hypovolemic shock due to leakage of plasma from the intravascular to the extravascular space. It is associated with hemoconcentration, hypoalbuminemia, and generalized edema. We report the case of a patient with idiopathic systemic capillary leak syndrome who developed an unexpected and potentially fatal abdominal and four-limb compartment syndrome. This was successfully treated with fasciotomies and medical treatment including terbutaline, theophylline, and corticosteroids. To the best of our knowledge this is the first report of this kind in the literature.

**Case presentation:**

A previously healthy 54-year-old Caucasian man presented to the emergency department of our internal medicine ward with a medical history of aggravation of general health related to dizziness, weight gain, and two syncopal attacks. Due to a massive emission of fluids and proteins from the intravascular to the extracellular compartments, he developed compartment syndromes in his upper and lower limbs and the abdominal compartment. The abdomen and all four limbs required decompression by a fasciotomy of both forearms, both thighs, both lower legs, and the abdomen within 24 hours after admission. After 60 days of treatment he was dismissed from the clinic. He was able to return to his previous occupation and reached the same level of athletic activity as before the illness.

**Conclusions:**

Systemic capillary leak syndrome is a very rare disease that can lead to a fatal clinical outcome. It is important to be aware of the fatal complications that can be caused by this disease. Despite the fact that systemic capillary leak syndrome represents a very rare disease it is still important to be aware of life threatening complications, like compartment syndromes, which need surgical intervention. However, early diagnosis and interdisciplinary treatment can lead to a good clinical outcome.

## Introduction

Idiopathic systemic capillary leak syndrome (SCLS), also known as Clarkson’s disease, and spontaneous periodic edema is a very rare and life-threatening disorder first described in 1960 by Clarkson *et al.*[1]. It is associated with transient, recurrent episodes of capillary hyperpermeability that lead to the seepage of fluids and proteins from the intravascular to the extravascular compartments. The clinical course is characterized by hypotensive shock, hypoalbuminemia occurring in the absence of albuminuria [2], hemoconcentration (indicated by an elevated hematocrit), and edema of the limbs [1]. The most common prodromal symptoms described are flu-like symptoms, abdominal pain, dizziness, nausea, and vomiting [3]. In this case report we report on the case of a patient with idiopathic SCLS who developed an abdominal and four-limb compartment syndrome which was successfully treated with fasciotomies and medical treatment including terbutaline, theophylline, and corticosteroids. To the best of our knowledge this is the first report that shows the combination of a four-limb compartment syndrome and an abdominal compartment that needed surgical intervention. With this case presentation we would like to increase the awareness of this very rare disease and its early surgical treatment.

## Case presentation

**Figure 1:**
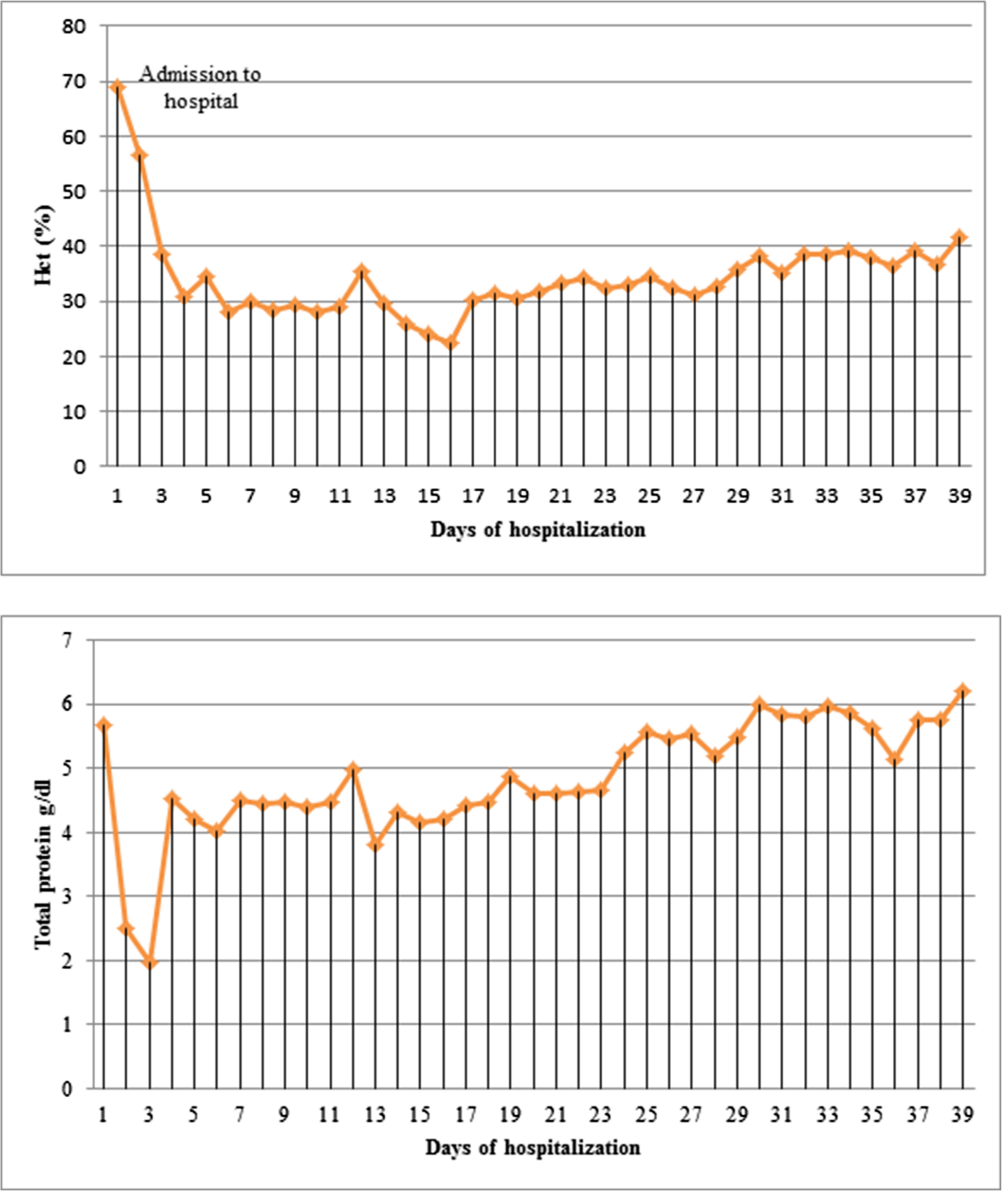
Hematocrit (HCT) and total protein values before and after fasciotomy of the compartment syndrome.

**Figure 2:**
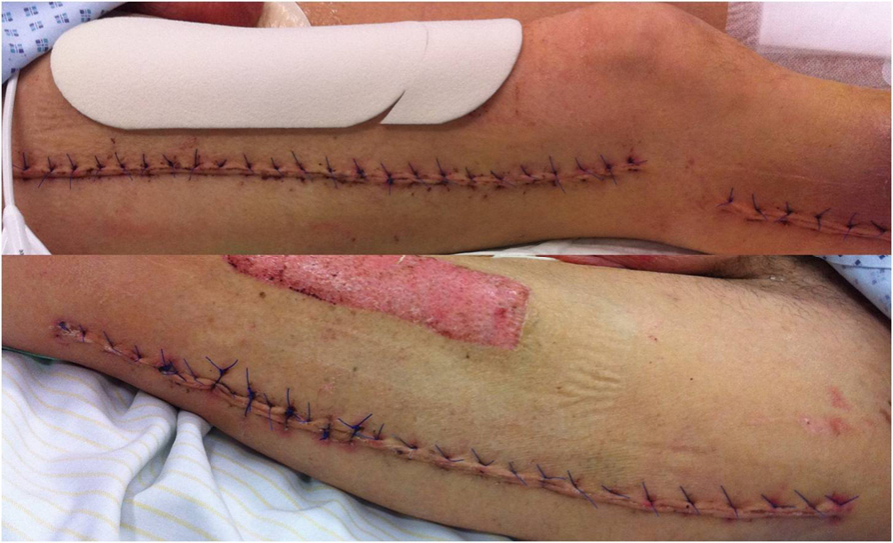
Closed wounds of the lower limbs (right/left) 10 days after wound closure.

**Figure 3:**
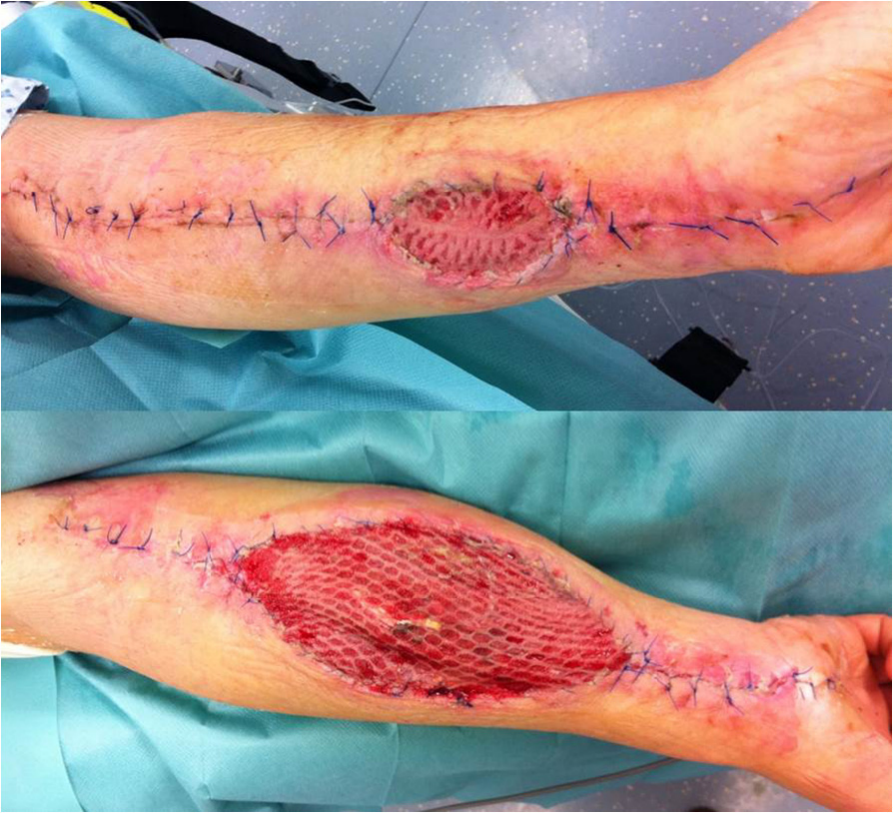
Closure of the upper limbs with skin grafting (right/left) 10 days after wound closure.

A previously healthy 54-year-old Caucasian man presented to the emergency department of our internal medicine ward with a medical history of aggravation of general health related to dizziness, weight gain, and two syncopal attacks. A physical examination was conducted which showed his blood pressure to be normotensive and his measured pulse rate and body temperature to be within normal limits. His initial laboratory results showed an increased hematocrit level of 69%, a hemoglobin level of 23g/dl, and a white blood cell count of 15.5×1000/μL. Initially he seemed to be hemodynamically stable with no signs of dyspnea. He experienced a rapid decrease of total proteins (5.67g/dl decreasing to 2.02g/dl within 72 hours) and began to exhibit hemodynamic instability, at which point he was admitted to our intensive care unit (ICU) and treated with catecholamines. Due to an increasing pulmonary insufficiency an endotracheal intubation was performed immediately. A massive emission of fluids and proteins from the intravascular to the extracellular compartments caused a generalized compartment syndrome to develop. Over 24 hours after admission to the ICU our patient developed compartment syndromes in both his upper and lower limbs and the abdominal compartment. The abdominal compartment syndrome was diagnosed by measuring the intra-abdominal pressure through a urinary catheter. The highest measured intra-abdominal pressure (IAP) was 26mm/Hg and therefore a diagnosis of abdominal compartment syndrome Grade IV was made. His abdomen and all four limbs required decompression by a fasciotomy of both forearms, both thighs, both lower legs, and the abdomen. The surgeries were performed 24 hours after admission to the clinic. Vacuum-assisted dressings were placed first on his lower limbs. During a second revision operation vacuum-assisted dressings were placed on his upper limbs to assist with monitoring the edema and in preparation for the definite closure of the fasciotomy wounds. The dressing of his abdomen included putting the intestine into a sac and covering it with a transparency dressing. Continuous renal replacement therapy (CRRT) was required three days after admission due to acute renal failure. Continuous venovenous hemodiafiltration (CVVHD) was applied for a total of four days. The blood levels of creatinine and urea returned to normal after three days of CVVHD and he gained back full renal function. Before CVVHD the highest creatinine level amounted to 1.4mg/dl and after renal replacement therapy (before discharge from the hospital) it decreased to 0.6mg/dl. The urea levels also decreased from 80mg/dl to 17mg/dl. The hematological parameters returned to their normal limits by the fourth day of admission (Figure 1). The clinical diagnostics included cultures of the blood, urine, stool, sputum, and intra-operative tissue samples which were all analyzed for aerobic and anaerobic bacteria, as well as for fungus. The results of the samples were all negative. After ruling out the differential diagnoses the diagnosis of a SCLS was confirmed, with secondary abdominal compartment and compartment syndromes in all four limbs. The secondary closure of the abdomen had been performed 16 days after admission and 23 days after admission we were able to remove the vacuum-assisted pumps and proceed in closing all wounds (Figure 2). His upper limbs required skin grafting (Figure 3). His lower limbs showed weakness in the dorsal flexion of the feet and toes, therefore peroneal splints were adjusted to his feet. His upper limbs showed residual deficits of fine motor skills, especially the left upper limb. These deficits had been improved with hand therapy. Our patient was moved to the rheumatology ward after 23 days in our ICU. He was started on medical prophylactic treatment with theophylline and terbutaline in combination with steroid therapy (prednisolone). During remission induction therapy the dose of theophylline ranged between 1200 and 1600mg per day in order to achieve serum concentrations between 20 and 25mg/dl. Before discharging him the theophylline dose was reduced to 1000mg/day. In order to obtain the remission advised to achieve peak serum concentrations between 10 and 20mg/dl, terbutaline was first given at a total dose of 20mg per day in divided doses. Before he was discharged the dose was reduced to 10mg per day. We recommended that he should continue to take theophylline and terbutaline for the rest of his life. Methylprednisolone was applied intravenously while in remission induction therapy at a dose of 40mg per day. After remission was induced the prednisolone was gradually reduced to 15mg/day. We recommended maintaining the gradual reduction of that dose.

After 60 days of treatment he was discharged from the clinic. He was able to return to his previous place of work and reached the same level of athletic activity as before the illness.

## Discussion

SCLS is a rare, potentially fatal disease, with a high mortality rate. It is characterized by hypoproteinemia, acute hypovolemia, and edema of the limbs [1,3,4], however the pathogenesis remains unclear. The capillary leakage leads to a shift of intravascular fluids into the interstitium and extravascular space. This shift may cause severe complications such as compartment syndrome of the limbs or the abdomen, as it did in this case. The most common clinical features described from several case reports are flu-like prodromes including nausea, vomiting, abdominal pain, and myalgia. Further symptoms observed in patients are polydipsia, dizziness, hypotension, generalized edema (as well as cerebral, pulmonary, macular or epiglottic edema), weight gain, pleural or pericardial effusion, and renal dysfunction that could end in renal failure [5]. Potential laboratory markers found in patients with SCLS are elevated white blood cell count, elevated hematocrit (>55%), low serum total protein and albumin, or monoclonal immunoglobulinopathy such as increased immunoglobulin G (IgG) kappa, IgG lambda, or immunoglobulin A (IgA) lambda levels [5-7]. Most of the patients in the reported cases were previously healthy, indicating that SCLS appears to belong to diseases with an idiopathic genesis. The differential diagnoses that need to be excluded before confirming the diagnosis of SCLS are overwhelming sepsis, dengue shock syndrome, chemotherapy, lymphoma, Sézary syndrome, hereditary angioedema due to C1 esterase deficiency, and carbon monoxide poisoning [5-8].

Most of the reported cases show that the patients were described as previously healthy as was our patient. This indicates that a predisposition for this disease does not yet exist. The mean age for the onset of the disease appeared to be at 47±13.1 years, with a female to male ratio of 1:1.4 [3]. The pathophysiology of SCLS is still unknown and the process of finding a possible etiopathogenesis appears to be complex.

## Conclusions

SCLS is very rare disease that can lead to a fatal clinical outcome. It is important to be aware of the complications that can be caused by this disease. Most of the patients with SCLS first present to an internal emergency department. Therefore we believe that internists should be aware of potential complications requiring surgery. As shown in our case report, an early interdisciplinary treatment is required for patients with SCLS, which may then lead to a good clinical outcome.

## Consent

Written informed consent was obtained from the patient for publication of this case report and accompanying images. A copy of the written consent is available for review by the Editor-in-Chief of this journal.

## Abbreviations

CRRT: Continuous renal replacement therapy; CVVHD: Continuous venovenous hemodiafiltration; IAP: Intra-abdominal pressure; ICU: Intensive care unit; IgA: Immunoglobulin A; IgG: Immunoglobulin G; SCLS: Systemic capillary leak syndrome.

## Competing interests

The authors declare that they have no competing interests.

## Authors’ contributions

HL, JPG, MH, PJ analyzed and interpreted the patient data and performed the surgery on the patient. HL, JPG, MB, MW, MH, JW, PJ have been involved in drafting the manuscript or revising it critically for important intellectual content. HL, JPG, MH, PJ wrote the manuscript. All authors read and approved the final manuscript.
